# Chitosan Nanoparticles Alleviate Salinity‐Induced Growth Inhibition in in Vitro Shoot Cultures of Tobacco ( *Nicotiana tabacum* L.)

**DOI:** 10.1002/pei3.70222

**Published:** 2026-10-11

**Authors:** Neelu Joshi, Amar Hundare

**Affiliations:** ^1^ School of Biotechnology & Bioinformatics D.Y. Patil Deemed To be University Navi Mumbai Maharashtra India; ^2^ Eric Young Orchid Foundation Jersey UK

**Keywords:** antioxidant enzymes, chitosan, nanoparticles, salinity, tobacco

## Abstract

Salinity stress is a major environmental constraint that limits plant growth and productivity by disrupting ionic balance, inducing osmotic stress, and promoting oxidative damage. Chitosan (Ch), a naturally derived biopolymer, enhances plant tolerance to abiotic stresses, but its effectiveness may be limited by poor solubility and reduced interaction with plant tissues. Chitosan nanoparticles (ChNP) may overcome these limitations through improved bioavailability and cellular interaction. This study evaluated the effectiveness of Ch and ChNP in alleviating salinity stress in in vitro shoot cultures of tobacco ( *Nicotiana tabacum* L.), a valuable model system. Shoot cultures maintained on shoot multiplication medium (Murashige and Skoog (MS) medium, 0.2 mg L^−1^ NAA (1‐Naphthaleneacetic acid) and 2.0 mg L^−1^ BAP (6‐Benzylaminopurine)) were cultured on medium containing 150 mM NaCl with or without 20 ppm Ch or ChNP for 30 days. Salinity stress significantly reduced shoot length, shoot number, and shoot fresh weight by 33.3%, 39.7%, and 40%, respectively, compared with non‐stressed shoots. Both Ch and ChNP alleviated these effects, with ChNP showing greater efficacy by increasing shoot length, shoot number, and shoot fresh weight by 3.6‐, 2.0‐, and 3.2‐fold, respectively, relative to untreated stressed shoots. ChNP also reduced hydrogen peroxide, superoxide radical, and malondialdehyde levels while enhancing catalase, superoxide dismutase, and peroxidase activities. Furthermore, ChNP increased chlorophyll a, chlorophyll b, total chlorophyll, and proline contents by 77.9%, 167.5%, 86.8% and 27%, respectively. These findings demonstrate that ChNP effectively enhanced salinity tolerance by strengthening physiological and biochemical stress‐response mechanisms in tobacco under in vitro conditions.

## 1 Introduction

Soil salinity is one of the most common abiotic stresses that limit crop productivity worldwide. At a cellular level, excess sodium and chloride ions disturb ionic equilibrium, cause osmotic imbalance and trigger overaccumulation of reactive oxygen species (ROS). The resultant oxidative burst causes loss of membrane integrity, protein function and photosynthetic efficiency and, in turn, cumulatively reduces plant physiological performance (Gupta and Huang 2014). To combat salt stress, plants possess natural defense mechanisms such as ion compartmentalization, osmotic homeostasis and antioxidant metabolism. However, these processes are generally inadequate to provide protection under persistent salinity conditions (Cannea and Padiglia 2025). Chitosan, a natural biopolymer, is a well‐known biostimulant, due to its ability to impart tolerance against various abiotic stresses, including salinity. In its bulk form, chitosan functions by balancing the antioxidant defense system (Safikhan et al. 2018), osmotic homeostasis (Özkurt and Bektaş 2022), and photosynthetic efficiency (Shen et al. 2024) under salinity conditions. However, its potency may be impaired on account of weak solubility and limited interaction with the plant tissues. These problems have been solved by the development of chitosan nanoparticles as they possess higher surface area and bioavailability over bulk chitosan, thereby enhancing uptake by the plant cells (Divya and Jisha 2017; Ingle et al. 2022).

Chitosan nanoparticles have been reported to enhance growth parameters more effectively than bulk formulations under salt stress conditions by strengthening the antioxidant system (Hassan et al. 2021) and photosynthetic efficiency (Alenazi et al. 2024). Majority of such studies have focused on whole plants or seedlings grown under greenhouse (Cao et al. 2025), botanical garden (Alenazi et al. 2024) or open field conditions (Ghasemi et al. 2017), with nanoparticles applied through foliar spray (Hassan et al. 2021), root drenching (Shen et al. 2024), or seed priming (Sen et al. 2020). These studies are impeded by soil variability, plant‐microbe interactions, and unstable environmental conditions, that may influence plant responses, making it difficult to perceive the direct physiological effect of ChNPs.

In vitro culture platforms are characterized by controlled chemical and physical environments including nutrient and plant growth regulator composition, light intensity and temperature, allowing to determine/study the effect of each of these parameters on plant growth. This characteristic of in vitro systems is especially advantageous for salt stress mitigation studies as a more accurate and reproducible assessment of plant responses to stress alleviating agents can be determined. Furthermore, it facilitates clearer insights into the mode of action of stress alleviating agents by minimizing complex environmental influences. In addition, in vitro systems can serve as rapid screening platforms before greenhouse or field evaluation. Despite these advantages, the use of tissue culture systems to assess the ameliorative effect of ChNPs remains scarce.

*Nicotiana tabacum L*. is an ideal model species for such investigations due to its high regenerative capacity and well‐characterized physiological and stress responses (Budzianowska 2009). It's in vitro culture system provides a reproducible platform for precisely examining the effectiveness of stress alleviating agents. Therefore, the present study aimed to evaluate the efficacy of chitosan nanoparticles in alleviating salinity stress in *N. tabacum* shoot cultures under controlled in vitro conditions. This study conducted a systematic comparative evaluation of bulk chitosan and its nanoforms on growth responses, photosynthetic pigment dynamics, oxidative stress, and antioxidant enzyme activities, to gain mechanistic insights into the protective effects of ChNP. By using a pre‐optimized concentration of chitosan/chitosan nanoparticles at one pre‐identified physiologically relevant NaCl concentration, this study conducted a focused comparison of their stress mitigating effects.

## 2 Materials and Methods

### 2.1 Preparation of Chitosan Nanoparticles

ChNPs were prepared following the ionic gelation method (Liu and Gao 2009). Different concentrations (0.2%–0.8% w/v, dissolved in 1% acetic acid) of chitosan (low molecular weight, 75%–85% deacetylated, Sigma‐Aldrich, Product No. 448869) and tripolyphosphate (TPP; 0.05%–0.25% w/v, aqueous) solutions mixed in different ratios (4:1–1:1) were tested for nanoparticle preparation in preliminary studies. Based on the results, the optimized concentration of chitosan (0.4%), TPP (0.25% w/v), and Ch:TPP ratio (3:1) were selected for the present work. TPP solution was added dropwise to chitosan solution (chitosan:TPP ratio of 3:1) while maintaining continuous stirring. The mixture was agitated for 6 h to obtain an opalescent suspension, and subsequently centrifuged at 10,000 × *g* at 4°C for 15 min. The pellet was rinsed with distilled water, lyophilized, and stored at 4°C till further use. The nanoparticle suspension was prepared following Li et al. (2008).

The synthesized ChNPs were characterized as described by Joshi et al. (2018). Briefly, the hydrodynamic diameter and size distribution (polydispersity index, PDI) were determined by dynamic light scattering (DLS), and the zeta potential was measured by electrophoretic light scattering, using a NanoBrook Omni instrument at IIT Bombay, India. Scanning electron microscopy (SEM) and transmission electron microscopy (TEM) of ChNP were performed at the Sophisticated Analytical Instrument Facility (SAIF), IIT Bombay, India. The ChNPs recorded an average diameter of 480 nm, a PDI of 0.267, zeta potential of 22.84 mV (Figure S1), and a spherical morphology under TEM and SEM (Figure S2 and Figure S3). The suspension remained stable for around 2 weeks when stored at 4°C.

### 2.2 Plant Material and Experimentations

Seeds of *Nicotiana tabacum* L. cv. “Anand 119” obtained from Central tobacco research institute, Hyderabad, were used for establishment of shoot cultures. In vitro grown seedling‐derived leaf explants were inoculated on shoot multiplication media (SMM) composed of 4.3 g L^−1^ Murashige and Skoog (MS) basal medium, 0.2 mg L^−1^ NAA (1‐Naphthaleneacetic acid) and 2.0 mg L^−1^ BAP (6‐Benzylaminopurine) (Joshi 2009). The obtained shoot buds were sub‐cultured thrice on fresh medium at intervals of 21 days for the purpose of multiplication. The cultures were maintained at a temperature of 25°C ± 2°C with a photoperiod of 16 h (45 μmol m^−2^ s^−1^). The cultures from the multiplication phase were utilized as the experimental material.

To optimize the salinity level and concentrations of Ch/ChNP for the present study, preliminary dose‐response experiments were conducted. Among the NaCl concentrations evaluated (50–200 mM), 150 mM reduced shoot growth by 35% without causing mortality. Among the Ch and ChNP concentrations tested (10–100 ppm), 20 ppm produced maximum shoot growth and biomass. Based on these results, supported by published literature (150 mM NaCl: Hassan et al. 2021; 25 ppm ChNP:Cao et al. 2025, 15 ppm Ch: Asghari‐ Zakaria et al. 2009, 25 ppm Ch:Golkar et al. 2019) and the objectives of the present study, 150 mM NaCl and 20 ppm Ch and ChNP were selected as the optimum experimental conditions for evaluating stress‐mitigation effects.

A cluster of shoots weighing approximately 0.5 g (containing ca. 7 shoots measuring 1.0 cm) was inoculated on SMM (control) and SMM containing: (i) 150 mM NaCl, (ii) 150 mM NaCl + Chitosan (20 ppm), and (iii) 150 mM NaCl + Ch‐NPs (20 ppm, fresh suspension). All the cultures were incubated at 25°C ± 2°C under 16 h of photoperiod (45 μmol m^−2^ s^−1^) provided by cool white, fluorescent tubes. The cultures were maintained on the respective treatments for 30 days and the observations on growth and biochemical attributes were recorded.

### 2.3 Growth Traits

Measurements were taken for fresh weight (FW, g), shoot length (cm), and the number of shoots for plantlets from both the control group and each treatment group. Biochemical assays were performed on plantlets cultivated under conditions (i) to (iii) as well as for the control group.

### 2.4 Photosynthetic Pigment Contents

Arnon's (1994) spectrophotometric method was used to determine the chlorophyll contents. 100 mg FW of shoots were ground in 8 mL 80% acetone (v/v). The concentration of chlorophyll a, chlorophyll b, and total chlorophyll was calculated using the following equation:

$$\text{Total chlorophyll}=20.2\left(A_{645}\;\right)+8.02\left(A_{663}\;\right)$$

$$\text{Chlorophyll}\;a=12.7\left(A_{663}\right)-2.69\left(A_{645}\right)$$

$$\text{Chlorophyll}\;b=22.9\left(A_{645}\right)-4.68\left(A_{663}\right)$$

The results were expressed as μg g^−1^ FW of the tissues.

### 2.5 Proline Content

Proline content was determined colorimetrically using the ninhydrin method (Marin et al. 2009). Shoot tissue (500 mg) was homogenized in 2 mL of 3% (w/v) sulfosalicylic acid and centrifuged at 1500 × *g* for 10 min. The supernatant was combined with glacial acetic acid and 8% acidic ninhydrin in a 1:1:1 ratio, heated at 100°C for 1 h, and the chromophore was extracted with toluene. Absorbance was measured at 520 nm, and proline concentration was quantified using a standard curve of L‐proline (0–100 μg mL^−1^). Results are expressed as μM g^−1^ FW.

### 2.6 Determination of Malondialdehyde (MDA) Content

The degree of lipid peroxidation was assessed by measuring MDA (Fu and Huang 2001). Tissue samples weighing 1 g were homogenized in 5 mL of 0.1% (w/v) TCA at 4°C before centrifugation at 5000 *g* for 10 min at 4°C. The reaction mixture included 500 μL of supernatant and 4 mL of 0.5% (w/v) thiobarbituric acid in 20% (w/v) TCA. After 30 min of incubation at 95°C in a shaking water bath, the process was stopped by rapidly cooling the tubes in an ice bath. The samples were centrifuged at 5000 *g* for 15 min, and the supernatant absorbance was measured at 532, 600, and 450 nm. To eliminate the determination error caused by soluble sugar, the MDA concentration (μmol/g FW) was computed using the following formula:

$$\mathrm{MDA}=6.45\;\left(A_{532}-A_{600}\right)-0.56\;\left(A_{450}\right)$$

The concentration of MDA was determined by applying an extinction coefficient of 155 mM^−1^ cm^−1^.

### 2.7 Determination of Hydrogen Peroxide (H_2_O_2_ ) and Superoxide Radical (O_2_ ^•–^) Concentration

Hydrogen peroxide content was determined spectrophotometrically at 350 nm following the potassium iodide method (Junglee et al. 2014), using a standard curve prepared with analytical‐grade H_2_O_2_ in 0.1% TCA. Results are expressed as μM g^−1^ FW.

The superoxide radical (O_2_^•‐^) production rate was measured spectrophotometrically by monitoring cytochrome c reduction at 550 nm (Doke 1983; Cosi et al. 2007), using a molar extinction coefficient of 2.1 × 10^4^ M^−1^ cm^−1^. Results are expressed as reduced Cyt c (μM min^−1^ g^−1^ FW).

### 2.8 Determination of CAT, POD and SOD Activities

Shoot tissue (200 mg) was powdered in liquid nitrogen and extracted in 2 mL of 100 mM potassium phosphate buffer (pH 7.5) containing 2.0 mM Na‐EDTA, 25% Triton X‐100, and 1.0% (w/v) PVP‐40. The homogenate was centrifuged at 10,000 × *g* for 15 min, and enzyme activities were determined in the supernatant according to Lowry et al. (1951). Catalase (CAT) activity was assayed by monitoring H_2_O_2_ consumption at 240 nm (Rao et al. 1996). Superoxide dismutase (SOD) activity was determined by the photochemical reduction of nitroblue tetrazolium (NBT) at 560 nm (Giannopolitis and Stanley 1977). Peroxidase (POD) activity was measured by monitoring guaiacol oxidation at 470 nm (Hammerschmidt et al. 1982). Enzyme activities are expressed as *U* mg^−1^ protein.

### 2.9 Statistical Analyses

The study utilized a randomized design with three replications for the experiments conducted (Table S1). The average values of the treatments and control were subjected to analysis of variance (ANOVA), which indicated significant differences as assessed by Duncan's Multiple Range Test (DMRT) at a significance level of 5%. The evaluation was performed utilizing IBM SPSS software (Version 22). Pearson correlation coefficients were computed for the entire dataset to investigate pairwise associations between physiological and biochemical markers (Table S2).

## 3 Results

### 3.1 Growth Traits

Salt stress significantly inhibited shoot growth, reducing shoot length by 33.3%, shoot number by 39.7%, and shoot fresh weight by 40% compared to non‐stressed controls (Table 1). Both Ch and ChNP mitigated these effects, with ChNP showing a stronger impact (Figure S4). ChNP treatment increased shoot length, shoot number, and fresh weight by 3.6‐, 2.0‐, and 3.2‐fold, respectively, relative to untreated stressed shoots, with the most pronounced improvement observed in shoot number and fresh weight.

**TABLE 1:** Effect of chitosan (Ch) and chitosan nanoparticles (ChNP) on growth, photosynthetic parameters and biochemical traits of *Nicotiana tabacum* shoots under in vitro salt stress (150 mM).

Treatment	Control	NaCl	NaCl+Ch	NaCl + ChNP
Shoot length (cm)	1.2 ± 0.1^c^	0.8 ± 0.1^c^	2.2 ± 0.1^b^	2.8 ± 0.1^a^
Number of Shoots	10.2 ± 0.1^b^	6.15 ± 0.1^d^	9.33 ± 0.2^c^	12.5 ± 0.2^a^
Total FW (g)	0.65 ± 0.03^c^	0.39 ± 0.03^d^	0.84 ± 0.03^b^	1.26 ± 0.04^a^
Chl a (μg g^−1^ FW)	11.24 ± 0.15^b^	6.87 ± 0.19^d^	10.41 ± 0.16^c^	12.21 ± 0.16^a^
Chl b (μg g^−1^ FW)	5.83 ± 0.11^a^	2.32 ± 0.10^c^	4.19 ± 0.15^b^	6.19 ± 0.16^a^
Total Chl (μg g^−1^ FW)	16.05 ± 0.16^b^	9.53 ± 0.10^d^	13.33 ± 0.18^c^	17.81 ± 0.16^a^
Proline (μM g^−1^ FW)	8.41 ± 0.26	15.87 ± 0.40^b^	16.42 ± 0.34^b^	20.13 ± 0.46^a^
H_2_O_2_ (μM g^−1^ FW)	47.33 ± 0.54^c^	70.56 ± 0.75^a^	55.56 ± 0.57^b^	45.12 ± 1.60^d^
O^2−^ Production rate (μM min^−1^g^−1^FW)	0.7 ± 0.07^b^	1.25 ± 0.12^a^	0.91 ± 0.08^b^	0.77 ± 0.07^b^
MDA (μM g^−1^ FW)	20.83 ± 0.26^c^	30.13 ± 0.46^a^	22.22 ± 0.34^b^	21.03 ± 0.31^bc^
CAT (μM min^−1^ mg^−1^ protein)	7.28 ± 0.12^d^	10.63 ± 0.23^c^	12.22 ± 0.17^b^	14.62 ± 0.26^a^
POD (U mg^−1^ protein)	8.43 ± 0.20^c^	13.74 ± 0.26^b^	14.21 ± 0.23^b^	19.22 ± 0.31^a^
SOD (U mg^−1^ protein)	14.46 ± 0.23^c^	18.23 ± 0.23^b^	18.95 ± 0.23^b^	20.34 ± 0.26^a^

*Note:* Values represent mean ± SE (*n* = 3). Means followed by different letters within a row indicate significant differences at *p* ≤ 0.05 according to Duncan's Multiple Range Test (DMRT).

Abbreviations: CAT, catalase; Chl, chlorophyll; FW, fresh weight; MDA, malondialdehyde; POD, peroxidase; SOD, superoxide dismutase.

### 3.2 Photosynthetic Pigments

Salt stress decreased photosynthetic pigments, with chlorophyll b showing the greatest reduction (60.2%). Both Ch and ChNP alleviated pigment loss, with ChNP being more effective, increasing chlorophyll a, chlorophyll b, and total chlorophyll contents by 77.9%, 167.5%, and 86.8%, respectively, compared to stressed shoots (Table 1).

### 3.3 Proline, ROS, and Lipid Peroxidation

Salt stress increased proline content by 88.7% (from 8.41 μM g^−1^ FW), while Ch and ChNP further elevated it to 16.42 μM g^−1^ FW and 26.8% above stressed shoots, respectively (Table 1). H₂O₂ and O_2_^•–^ levels rose by 50% and 78% under stress, whereas Ch reduced these by~21% and~27%, respectively, and ChNP reduced them by 36.1% and 38.4%, respectively. MDA content, a marker of membrane damage, increased by 44.6% under stress; ChNP reduced MDA by 30.2%, showing superior protective effects compared to Ch.

### 3.4 Enzymatic Antioxidant Activities

Salt stress enhanced CAT, SOD, and POD activities by 46.0%, 26.1%, and 63%, respectively. Ch treatment modestly increased these activities (CAT 15%, SOD 4%, POD 3.4%), whereas ChNP significantly boosted them by 37.5%, 11.6%, and 40.7% relative to untreated stressed shoots (Table 1).

## 4 Discussion

Under the imposed salinity regime (150 mM NaCl), tobacco shoot cultures exhibited marked growth inhibition, manifesting as reduced shoot elongation, diminished shoot proliferation, and lower biomass accumulation. Supplementation with either bulk chitosan or chitosan nanoparticles attenuated these adverse effects, with the nanoformulation consistently producing more pronounced amelioration. The growth‐promoting effect of chitosan aligns with previous reports in *Ocimum ciliatum* and *Ocimum basilicum* (Ghasemi et al. 2017) and *Phaseolus vulgaris* (Zayed et al. 2017). This effect is likely mediated through regulation of metabolic pathways, including carbon and nitrogen metabolism (Zhang et al. 2017), photosynthesis, plant growth regulator synthesis (Safikhan et al. 2018), enhanced nutrient uptake and stimulation of cell division and elongation, xylem development, and stomatal closure (Aazami et al. 2023). Importantly, the efficacy of chitosan is concentration‐dependent, with lower concentrations (25–50 mg/L) being more effective in mitigating stress‐related damage (Cao et al. 2025).

Despite the benefits of Ch, its solubility and stability can limit performance. Nanoformulations overcome these constraints, providing higher surface area, improved solubility, controlled release, and enhanced penetration into plant cells (Divya and Jisha 2017; Sathiyabama and Manikandan 2021; El‐Saadony et al. 2022). In agreement with our findings, ChNP more effectively enhanced shoot growth parameters under salinity compared to bulk chitosan, as reported in mung bean (Sen et al. 2020), and *Catharanthus roseus* (Hassan et al. 2021).

Studies have demonstrated a close link between growth and photosynthesis, and one of the most important indicators of efficient photosynthesis is concentrations of chlorophyll pigments. In the present study, both Ch and ChNP treatments significantly restored the salt‐induced decline of chlorophyll a, chlorophyll b, and total chlorophyll levels, with ChNP being more effective. Similarly, Mosavikia et al. (2020) demonstrated improved pigment synthesis in chitosan‐treated milk thistle under stress. Our results support the claim that chitosan improves the growth of stressed plants by increasing photosynthetic pigments (Li et al. 2008). This effect may be attributed to mechanisms such as the upregulation of enzymes involved in chlorophyll biosynthesis, stimulation of cytokinin‐mediated chlorophyll synthesis, or inhibition of chlorophyll‐degrading enzymes such as chlorophyllase (Li et al. 2008).

Proline accumulation is a key adaptive response to osmotic stress. Salt‐stressed tobacco shoots showed elevated proline, which was further enhanced by Ch and ChNP, with the latter showing the highest increase (26.83%) over stressed shoots. This indicates superior osmoprotective capacity of ChNP. Comparable results were observed in faba beans (Dawood et al. 2024) and hibiscus (Cao et al. 2025). Similarly, Alenazi et al. (2024) demonstrated that ChNP more effectively reduced electrolyte leakage and enhanced proline accumulation than bulk chitosan in *Phaseolus vulgaris* under salinity stress, corroborating the superior membrane protective and osmoprotective capacity of the nanoformulation observed in the present study.

Oxidative stress markers, H_2_O_2_, O_2_^•–^, and MDA increased under salinity, indicating membrane and oxidative damage. Both Ch and ChNP significantly reduced these levels, with ChNP being more effective. Chitosan's ROS‐scavenging capacity has been reported in milk thistle (Safikhan et al. 2018), sorghum (Mulaudzi et al. 2022), and pummelo (Nie et al. 2020). Mechanistically, chitosan interacts via its amine and hydroxyl groups with cellular components to stabilize membranes and reduce lipid peroxidation (Rabêlo et al. 2019). Additionally, chitosan can induce controlled H_2_O_2_ production as a signaling molecule, enhancing stress tolerance through improved growth and photosynthetic performance (Pongprayoon et al. 2013).

Antioxidant enzyme activities (CAT, SOD, POD) were significantly elevated under salinity, reflecting a natural defense response. Ch and ChNP further enhanced these activities, with ChNP inducing greater increases (37%–41% for CAT and POD). Chitosan interacts with plant receptors such as chitin elicitor binding proteins (CEBiP), activating stress signaling pathways mediated by ROS, H_2_O_2_, Ca^2+^, NO, and phytohormones, leading to upregulation of antioxidant metabolism genes (Rayanoothala et al. 2024). Nano‐chitosan has been shown to stimulate activities of CAT, SOD, POD, and glutathione reductase in diverse crops under abiotic stress (Alenazi et al. 2024). Moreover, Ch can upregulate stress‐responsive genes such as geissoschizine synthase, octadecanoid‐responsive AP2‐domain genes, and mitogen‐activated protein kinases, contributing to enhanced stress tolerance (Hassan et al. 2021).

In the present study, the overall physiological condition was measured at one time point (30 days). Antioxidant enzymes are known to show time‐course changes during the stress period, including brief spikes and regulatory changes that would be missed by a single end‐point measurement. Our results provide a snapshot of integrated, steady‐state antioxidant capability under extended salinity, but time‐course investigations can give further insight into the kinetics of enzyme induction. The higher effectiveness of ChNP than bulk Ch is assumed to be related to increased cellular uptake via endocytosis or pores, increased interaction with cell wall components and membranes due to higher surface reactivity, and increased activation of chitin elicitor binding proteins (CEBiP) that activate ROS, Ca^2+^, NO and phytohormone signaling pathways (Rayanoothala et al. 2024). Putative pathways are inferred from physiological and biochemical endpoints examined in the current study. Direct molecular evidence such as gene expression analysis of antioxidant and osmolyte production genes was not acquired and is an essential avenue for future research.

Pairwise interactions between growth, photosynthetic, oxidative stress, and antioxidant parameters were examined across all observations (*n* = 12) using Pearson correlation analysis (Table S2). The antioxidant enzymes CAT, POD, and SOD showed strong positive relationships with proline concentration (*r* > 0.98, *p* < 0.001) and with each other (*r* > 0.96, *p* < 0.001), indicating a coordinated induction of both enzymatic and non‐enzymatic defense mechanisms under salinity. Restoration of photosynthetic capacity is closely linked to biomass recovery (Jiao et al. 2024), as evidenced by the strong positive correlation between photosynthetic pigments (chlorophyll a, chlorophyll b, and total chlorophyll) and growth parameters such as shoot number, shoot length, and fresh weight (*r* > 0.95, *p* < 0.001) in our study. On the other hand, oxidative stress markers (H_2_O_2_ and MDA) were positively correlated with each other (*r* = 0.96, *p* < 0.001) and strongly negatively correlated with photosynthetic and growth parameters (*r* = −0.94 to −0.97, *p* < 0.001), which is consistent with the theory that ROS accumulation and lipid peroxidation limit growth performance under salt stress (Alenazi et al. 2024). When taken as a whole, these correlation patterns support a stress‐alleviation sequence in which treatment with chitosan nanoparticles increases proline accumulation and antioxidant activity, lowering oxidative damage and maintaining photosynthetic function, which is linked to increased biomass and shoot growth. These connections confirm the greater effectiveness of chitosan nanoparticles over bulk chitosan in reducing salt stress and offer an integrated physiological viewpoint that enhances the individual treatment comparisons.

Important to note out that in this study, Ch and ChNP were added to the culture medium concurrently with salt stress, and the plants were continuously exposed to them for the 30 days of the experiment. This is contrary to several other studies in which chitosan preparations were applied as a priming agent before stress exposure (Sen et al. 2020) causing preconditioning or systemic acquired resistance. Continuous exposure to stress relieving agents ensures their bioavailability and direct alleviation of ongoing ionic and osmotic stress, whereas priming may activate additional signaling pathways and stress responses. This procedural variation must be considered when comparing ameliorative results reported by different studies.

Unlike most of the prior works, the current work evaluates the impacts of chitosan nanoparticles in an in vitro growth system rather than under greenhouse or field environments. In this system, the experimental conditions were controlled with defined nutrient composition, salinity level, and optimized concentrations of chitosan and chitosan nanoparticles, thus minimizing environmental variability and providing a direct assessment of their physiological and biochemical effects. These systems are valuable for mechanistic investigations and for fast screening of stress‐mitigating chemicals before large‐scale validation. However, the controlled nature of the research is also a limitation of the current study, since it does not completely duplicate the complexity of greenhouse/field environments, where elements such as soil variability, microbial interactions, and climatic changes may affect plant responses. Therefore, the straightforward extrapolation of the current results to field settings should be treated with care, and confirmation under field conditions is necessary before practical agricultural suggestions can be made.

## 5 Conclusions

Chitosan nanoparticles were more effective than bulk chitosan in relieving NaCl‐induced growth inhibition of in vitro shoot cultures of *N. tabacum* . The most prominent increase was in shoot length and fresh weight with increased chlorophyll levels. ChNP treatment exhibited decreased hydrogen peroxide, superoxide radical, and malondialdehyde concentrations and higher antioxidant enzyme activity indicating less oxidative and membrane damage. Proline levels were increased also with ChNP therapy suggesting better osmotic adjustment. These findings demonstrate that chitosan nanoparticles are useful for enhancing the physiological and biochemical stress‐response systems to improve salt tolerance in vitro circumstances. Further validation under greenhouse and field settings is needed before ChNP can be proposed as a feasible agricultural technique to improve plant resistance under salt stress.

## Funding

The authors have nothing to report.

## Conflicts of Interest

The authors declare no conflicts of interest.

## Supporting information


**Figure S1:** Zeta Potential (22.84 mV) of chitosan nanoparticles.
**Figure S2:** Size distribution of chitosan nanoparticles.
**Figure S3:** TEM and SEM images of chitosan nanoparticles.
**Figure S4:** Effect of Chitosan nanoparticle on growth of Nicotiana Tabacum L. shoots under in vitro salinity stress: (a) control (unstressed), (b) 150 mM NaCl, (c) 150 mM NaCl+ Ch, (d) 150 mM NaCl+ ChNP.
**Table S1:** Raw data of growth, photosynthetic, oxidative stress, and antioxidant parameters measured in in vitro shoot cultures of Nicotiana tabacum L. under control, 150 mM NaCl, 150 mM NaCl +20 ppm chitosan (Ch), and 150 mM NaCl +20 ppm chitosan nanoparticles (ChNP) treatments. Values represent individual replicate observations (*n* = 3 per treatment).
**Table S2:** Pairwise Pearson correlation coefficients among growth, photosynthetic, oxidative stress, and antioxidant parameters in in vitro shoot cultures of Nicotiana tabacum L. across all treatments (*n* = 12). Significance levels: *** *p* < 0.001, ** *p* < 0.01, * *p* < 0.05; ns = not significant. Strength classifications follow conventional descriptors: Very Strong (|r| ≥ 0.90), Strong (0.70 ≤ |r| < 0.90), Moderate (0.40 ≤ |r| < 0.70), Weak (|r| < 0.40).

## Data Availability

The data that supports the findings of this study are available in the supporting information of this article.
