# Supplementary material for: Chitosan Nanoparticles Alleviate Salinity‐Induced Growth Inhibition in in Vitro Shoot Cultures of Tobacco ( Nicotiana tabacum L.)
Source: Plant Environ Interact. 2026 Oct 11;7(5):e70222. doi: 10.1002/pei3.70222 (PMC13655362; doi:10.1002/pei3.70222)
Supplement: Supplementary file 1 [file PEI3-7-e70222-s001.docx]

**Figure S1:** Zeta Potential (22.84 mV) of chitosan nanoparticles

# **Figure S2:** Size distribution of chitosan nanoparticles

**Figure S3:** TEM and SEM images of chitosan nanoparticles

**Figure S4:** Effect of Chitosan nanoparticle on growth of *Nicotiana Tabacum* L. shoots under *in vitro* salinity stress: a) control (unstressed), b) 150 mM NaCl, c) 150 mM NaCl+ Ch, d) 150 mM NaCl+ ChNP

**Table S1.** Raw data of growth, photosynthetic, oxidative stress, and antioxidant parameters measured in in vitro shoot cultures of *Nicotiana tabacum* L. under control, 150 mM NaCl, 150 mM NaCl + 20 ppm chitosan (Ch), and 150 mM NaCl + 20 ppm chitosan nanoparticles (ChNP) treatments. Values represent individual replicate observations (n = 3 per treatment).

| **Treatment** | **Control** | **Control** | **Control** | **NaCl** | **NaCl** | **NaCl** | **NaCl+Ch** | **NaCl+Ch** | **NaCl+Ch** | **NaCl+ChNP** | **NaCl+ChNP** | **NaCl+ChNP** |
| --- | --- | --- | --- | --- | --- | --- | --- | --- | --- | --- | --- | --- |
| **Replicate** | **1** | **2** | **3** | **1** | **2** | **3** | **1** | **2** | **3** | **1** | **2** | **3** |
| Shoot length (cm) | 1 | 1.2 | 1.4 | 0.58 | 0.8 | 1.02 | 1.95 | 2.25 | 2.5 | 2.58 | 2.85 | 3.12 |
| Number of Shoots | 10 | 10.2 | 10.4 | 6 | 6.3 | 6.4 | 9 | 9.3 | 9.7 | 12.1 | 12.5 | 12.9 |
| Total FW (g) | 0.6 | 0.63 | 0.72 | 0.34 | 0.38 | 0.45 | 0.78 | 0.85 | 0.89 | 1.18 | 1.26 | 1.34 |
| Chl a (µg g^-1^ FW) | 10.9 | 11.4 | 11.35 | 6.5 | 6.95 | 7.15 | 10.1 | 10.45 | 10.65 | 11.9 | 12.25 | 12.45 |
| Chl b (µg g^-1^ FW) | 5.6 | 5.8 | 6 | 2.15 | 2.35 | 2.5 | 3.9 | 4.15 | 4.45 | 5.9 | 6.25 | 6.45 |
| Total Chl (µg g^-1^ FW) | 15.8 | 16 | 16.35 | 9.35 | 9.55 | 9.7 | 13 | 13.35 | 13.65 | 17.5 | 17.85 | 18.08 |
| Proline (µM g^-1^ FW) | 8 | 8.3 | 8.9 | 15.2 | 15.8 | 16.6 | 15.8 | 16.4 | 17 | 19.3 | 20.1 | 20.9 |
| H2O2 (µM g^-1^ FW) | 46.4 | 47.3 | 48.3 | 69.2 | 70.6 | 71.8 | 54.5 | 55.6 | 56.5 | 44 | 45.2 | 46.1 |
| O^2-^ rate (µM min^-1^g^-1^FW) | 0.57 | 0.7 | 0.83 | 1.03 | 1.25 | 1.47 | 0.77 | 0.91 | 1.06 | 0.63 | 0.76 | 0.89 |
| MDA (µM g^-1^ FW) | 20.4 | 20.8 | 21.3 | 29.3 | 30.2 | 30.9 | 21.6 | 22.3 | 22.8 | 20.5 | 21.1 | 21.6 |
| CAT (µM min^-1^ mg^1^protein) | 7 | 7.4 | 7.3 | 10.3 | 10.7 | 11.1 | 11.9 | 12.3 | 12.5 | 14.2 | 14.7 | 15.1 |
| POD (U mg^-1^ protein) | 8.1 | 8.5 | 8.8 | 13.3 | 13.8 | 14.2 | 13.8 | 14.3 | 14.6 | 18.7 | 19.3 | 19.8 |
| SOD (U mg^-1^ protein) | 14.1 | 14.6 | 14.9 | 17.9 | 18.3 | 18.7 | 18.6 | 19 | 19.3 | 19.9 | 20.4 | 20.8 |

**Table S2.** Pairwise Pearson correlation coefficients among growth, photosynthetic, oxidative stress, and antioxidant parameters in in vitro shoot cultures of Nicotiana tabacum L. across all treatments (n = 12). Significance levels: *** p < 0.001, ** p < 0.01, * p < 0.05; ns = not significant. Strength classifications follow conventional descriptors: Very Strong (|r| ≥ 0.90), Strong (0.70 ≤ |r| < 0.90), Moderate (0.40 ≤ |r|

< 0.70), Weak (|r| < 0.40).

| **Parameter 1** | **Parameter 2** | **r** | **p-value** | **Significance** | **Direction** | **Strength** |
| --- | --- | --- | --- | --- | --- | --- |
| CAT | POD | 0.9839 | 0 | *** | Positive | Very Strong |
| CAT | Proline | 0.9792 | 0 | *** | Positive | Very Strong |
| Chl | SN | 0.9718 | 0 | *** | Positive | Very Strong |
| CAT | SOD | 0.9782 | 0 | *** | Positive | Very Strong |
| Chl a | Chl b | 0.9675 | 0 | *** | Positive | Very Strong |
| Chl a | H2O2 | -0.9679 | 0 | *** | Negative | Very Strong |
| Chl a | Total Chl | 0.973 | 0 | *** | Positive | Very Strong |
| Chl b | H2O2 | -0.9701 | 0 | *** | Negative | Very Strong |
| Chl b | Total Chl | 0.9926 | 0 | *** | Positive | Very Strong |
| POD | Proline | 0.9785 | 0 | *** | Positive | Very Strong |
| SN | Total Chl | 0.9805 | 0 | *** | Positive | Very Strong |
| H2O2 | Total Chl | -0.9746 | 0 | *** | Negative | Very Strong |
| SL | Total FW | 0.9645 | 0 | *** | Positive | Very Strong |
| Proline | SOD | 0.9956 | 0 | *** | Positive | Very Strong |
| H2O2 | MDA | 0.9621 | 0.000001 | *** | Positive | Very Strong |
| Chl b | SN | 0.9563 | 0.000001 | *** | Positive | Very Strong |
| POD | SOD | 0.9628 | 0.000001 | *** | Positive | Very Strong |
| Chl | MDA | -0.9425 | 0.000005 | *** | Negative | Very Strong |
| H2O2 | SN | -0.9393 | 0.000006 | *** | Negative | Very Strong |
| SN | Total FW | 0.9153 | 0.00003 | *** | Positive | Very Strong |
| Chl b | MDA | -0.8929 | 0.000092 | *** | Negative | Strong |
| MDA | Total Chl | -0.8928 | 0.000093 | *** | Negative | Strong |
| MDA | O^2-^Rate | 0.889 | 0.00011 | *** | Positive | Strong |
| H2O2 | O^2-^Rate | 0.8805 | 0.000156 | *** | Positive | Strong |
| MDA | SN | -0.8652 | 0.000278 | *** | Negative | Strong |
| Chl | Total FW | 0.8381 | 0.000664 | *** | Positive | Strong |
| Total Chl | Total FW | 0.8209 | 0.001064 | ** | Positive | Strong |
| SN | SL | 0.8093 | 0.001429 | ** | Positive | Strong |
| CAT | SL | 0.7911 | 0.002182 | ** | Positive | Strong |
| O^2-^Rate | Total Chl | -0.7698 | 0.00341 | ** | Negative | Strong |
| Chl b | Total FW | 0.7618 | 0.003981 | ** | Positive | Strong |
| Chl b | O^2-^Rate | -0.759 | 0.004202 | ** | Negative | Strong |
| Chl | O^2-^Rate | -0.7535 | 0.004654 | ** | Negative | Strong |
| Chl | SL | 0.7495 | 0.005013 | ** | Positive | Strong |
| H2O2 | Total FW | -0.7448 | 0.005446 | ** | Negative | Strong |
| POD | SL | 0.7357 | 0.006387 | ** | Positive | Strong |
| CAT | Total FW | 0.714 | 0.009099 | ** | Positive | Strong |
| SN | O^2-^Rate | -0.7033 | 0.010721 | * | Negative | Strong |
| POD | Total FW | 0.6938 | 0.012319 | * | Positive | Moderate |

| MDA | Total FW | -0.6817 | 0.014622 | * | Negative | Moderate |
| --- | --- | --- | --- | --- | --- | --- |
| SL | Total Chl | 0.6814 | 0.014678 | * | Positive | Moderate |
| SOD | SL | 0.6763 | 0.015746 | * | Positive | Moderate |
| Proline | SL | 0.6623 | 0.018957 | * | Positive | Moderate |
| Chl b | SL | 0.6196 | 0.031642 | * | Positive | Moderate |
| H2O2 | SL | -0.6098 | 0.035252 | * | Negative | Moderate |
| MDA | SL | -0.5987 | 0.039712 | * | Negative | Moderate |
| Proline | Total FW | 0.5734 | 0.051265 | ns | Positive | Moderate |
| SOD | Total FW | 0.5673 | 0.054383 | ns | Positive | Moderate |
| O^2-^Rate | Total FW | -0.4701 | 0.123011 | ns | Negative | Weak |
| CAT | SN | 0.3814 | 0.22122 | ns | Positive | Weak |
| SN | POD | 0.3751 | 0.22962 | ns | Positive | Weak |
| O^2-^Rate | SL | -0.323 | 0.305842 | ns | Negative | Weak |
| O^2-^Rate | SOD | 0.2948 | 0.352189 | ns | Positive | Weak |
| O^2-^Rate | Proline | 0.2781 | 0.381453 | ns | Positive | Weak |
| CAT | Chl | 0.2294 | 0.473202 | ns | Positive | Weak |
| SN | Proline | 0.2086 | 0.515234 | ns | Positive | Weak |
| POD | Total Chl | 0.2074 | 0.517837 | ns | Positive | Weak |
| CAT | Total Chl | 0.2015 | 0.530089 | ns | Positive | Weak |
| Chl | POD | 0.1947 | 0.544347 | ns | Positive | Weak |
| SN | SOD | 0.1941 | 0.545627 | ns | Positive | Weak |
| MDA | Proline | 0.1536 | 0.633545 | ns | Positive | Weak |
| MDA | SOD | 0.1447 | 0.653694 | ns | Positive | Weak |
| O^2-^Rate | POD | 0.1234 | 0.702313 | ns | Positive | Weak |
| H2O2 | SOD | 0.1091 | 0.73583 | ns | Positive | Weak |
| Chl b | POD | 0.1056 | 0.744043 | ns | Positive | Weak |
| CAT | O^2-^Rate | 0.1053 | 0.744573 | ns | Positive | Weak |
| CAT | Chl b | 0.1004 | 0.756175 | ns | Positive | Weak |
| H2O2 | Proline | 0.096 | 0.76658 | ns | Positive | Weak |
| CAT | H2O2 | -0.0935 | 0.772512 | ns | Negative | Weak |
| Chl b | SOD | -0.0915 | 0.777427 | ns | Negative | Weak |
| H2O2 | POD | -0.0784 | 0.808722 | ns | Negative | Weak |
| Chl b | Proline | -0.0741 | 0.818941 | ns | Negative | Weak |
| CAT | MDA | -0.0464 | 0.886177 | ns | Negative | Weak |
| Chl | SOD | 0.04 | 0.901687 | ns | Positive | Weak |
| Chl | Proline | 0.0394 | 0.903155 | ns | Positive | Weak |
| Proline | Total Chl | 0.0263 | 0.935388 | ns | Positive | Weak |
| MDA | POD | 0.0139 | 0.965795 | ns | Positive | Weak |
| SOD | Total Chl | 0.0065 | 0.984001 | ns | Positive | Weak |

Abbreviations: SL = SL; SN = SN; FW = fresh weight; Chl = chlorophyll; MDA = malondialdehyde; CAT = catalase; POD = peroxidase; SOD = superoxide dismutase.
